# Clinical significance of post-surgical residual tumor burden and radiation therapy in treating patients with lacrimal adenoid cystic carcinoma

**DOI:** 10.18632/oncotarget.10259

**Published:** 2016-06-23

**Authors:** Jae Myoung Noh, Eonju Lee, Yong Chan Ahn, Dongryul Oh, Yoon-Duck Kim, Kyung In Woo, Young-Hyeh Ko, Seokhwi Kim

**Affiliations:** ^1^ Department of Radiation Oncology, Samsung Medical Center, Sungkyunkwan University School of Medicine, Seoul, Republic of Korea; ^2^ Department of Ophthalmology, Samsung Medical Center, Sungkyunkwan University School of Medicine, Seoul, Republic of Korea; ^3^ Department of Pathology, Samsung Medical Center, Sungkyunkwan University School of Medicine, Seoul, Republic of Korea; ^4^ Department of Radiation Oncology, Samsung Changwon Hospital, Changwon, Republic of Korea

**Keywords:** adenoid cystic carcinoma, lacrimal gland, surgery, radiation therapy

## Abstract

Retrospective analyses were done on 19 lacrimal adenoid cystic carcinoma (ACC) patients who underwent curative treatment between 1997 and 2013. Nine patients (47.4%) had T1-2 disease and ten (52.6%) had T4 disease. Surgical procedures were globe-preserving tumor resection in 11 patients (57.9%), incisional biopsy in five (26.3%), and orbital exenteration was undertaken in three (15.8%). Residual tumor burdens were R0/1 in 12 patients (63.2%) and R2 in seven (36.8%). Radiation therapy (RT) was recommended to all patients, and 16 (84.2%) completed RT (median 60 Gy). After median follow-up of 57.5 months, seven (36.8%) developed progression and three (15.8%) died. Local recurrence occurred in four patients (21.1%), distant metastasis in one (5.3%), and combined local recurrence and distant metastasis in two (10.5%). Progression-free survival and overall survival rates at 5-years were 64.5% and 82.6%, respectively. Among 12 patients following R0/1 resection, two (16.7%) developed local recurrence and none died, while among seven following R2 resection, five (71.4%) developed progression and three (42.9%) died. RT following R0/R1 resection could reduce progression. Globe-preserving surgery and RT seemed optimal strategy for T1-2 disease. Careful attention should to be paid to minimize residual tumor burden at surgery and effort for safe radiation dose escalation would be desired.

## INTRODUCTION

Adenoid cystic carcinoma (ACC) is a rare, slow-growing malignancy of the secretory glands with a prolonged clinical course [1-5]. According to the Surveillance, Epidemiology, and End Results (SEER) database, ACC rising in the eye and orbit accounted for only 1.8% of total patients, among who the lacrimal gland was most commonly involved in over 80%, and was associated with poor survival [5]. The seventh edition of the American Joint Committee on Cancer (AJCC) Staging Manual has defined T stage based on tumor size and extension to surrounding tissues [6, 7] (Table 1).

Defining a single best treatment strategy has been difficult because of the rarity of lacrimal ACC. In fact, most previous reports included only a limited number of patients, and, moreover, most patients had quite heterogeneous clinical features [8]. Surgical resection has been the most important and orbital exenteration has been commonly recommended to the patients with T3-4 disease [5]. Based on the invasiveness of primary tumor and the surgical extent, post-operative radiation therapy (RT) has been commonly recommended [7-10]. The clinical outcomes following aggressive treatment approaches, however, have not been satisfactory [8]. Moreover, the role of RT in relation to the post-surgical residual tumor burden has not been addressed yet.

In the current study, we retrospectively reviewed the treatment outcomes of lacrimal ACC patients accrued in a single institute to evaluate the prognostic influences of histological subtype, surgical extent, residual tumor burden, and role of RT.

**Table 1:** Definition of T stage of lacrimal gland tumors (AJCC 7^th^ edition)

T1		Tumor 2 cm or smaller in greatest dimension, with or without extraglandular extension into the orbital soft tissue
T2*		Tumor larger than 2 cm but not larger than 4 cm in greatest dimension
T3*		Tumor larger than 4 cm in greatest dimension
T4		Tumor invades periosteum or orbital bone or adjacent structures
	T4a	Tumor invades periosteum
	T4b	Tumor invades orbital bone
	T4c	Tumor invades adjacent structures (brain, sinus, pterygoid fossa, temporal fossa)

* Because the maximum size of the lacrimal gland in 2 cm, T2 and greater tumors will usually extend into the orbital soft tissue.

## RESULTS

The median age of all patients was 40 years (17∼64 years), and there were ten female and nine male. Nine patients (47.4%) had T1-2 disease and ten (52.6%) had T4 disease, respectively. The reasons for T4 stage assignment were bone invasion (T4b) in six patients and intracranial extension (T4c) in four. Twelve patients (63.2%) had tumors of 3 cm or larger and seven (36.8%) had tumors smaller than 3 cm. The patients’ characteristics are summarized in Table 2.

The types of surgery were orbital exenteration in three patients (15.8%), globe-preserving apparent gross tumor resection in 11 (57.9%), and incisional biopsy only in five (26.3%), respectively (Tables 2, 3). The residual tumor burden following surgery was assessed based on the surgical records, pathological findings and post-operative imaging: one patient (5.3%) had R0 (no evidence of residual lesion); 11 (57.9%) had R1 (microscopic involvement of the resection margin without grossly remaining lesion); and seven (36.8%) had R2 (either unresected gross tumor or radiographic evidence of gross residual lesion), respectively. Among four T4c patients, three underwent biopsy only while one did orbital exenteration.

Though RT was recommended to all patients, 16 (84.2%) could complete RT, two (10.5%) refused RT following R1 resection, and one (5.3%) had to discontinue RT at 16 Gy because of wound problem following orbital exenteration. The median total dose delivered to 16 patients was 60 Gy (59.4∼70 Gy) by daily doses ranging from 2.0 Gy to 2.3 Gy. RT techniques used were 3-dimensional conformal RT in 12 patients (70.6%) and intensity-modulated RT by Helical Tomotherapy in five (29.4%). No one received planned chemotherapy in relation to the current treatment.

**Figure 1:**
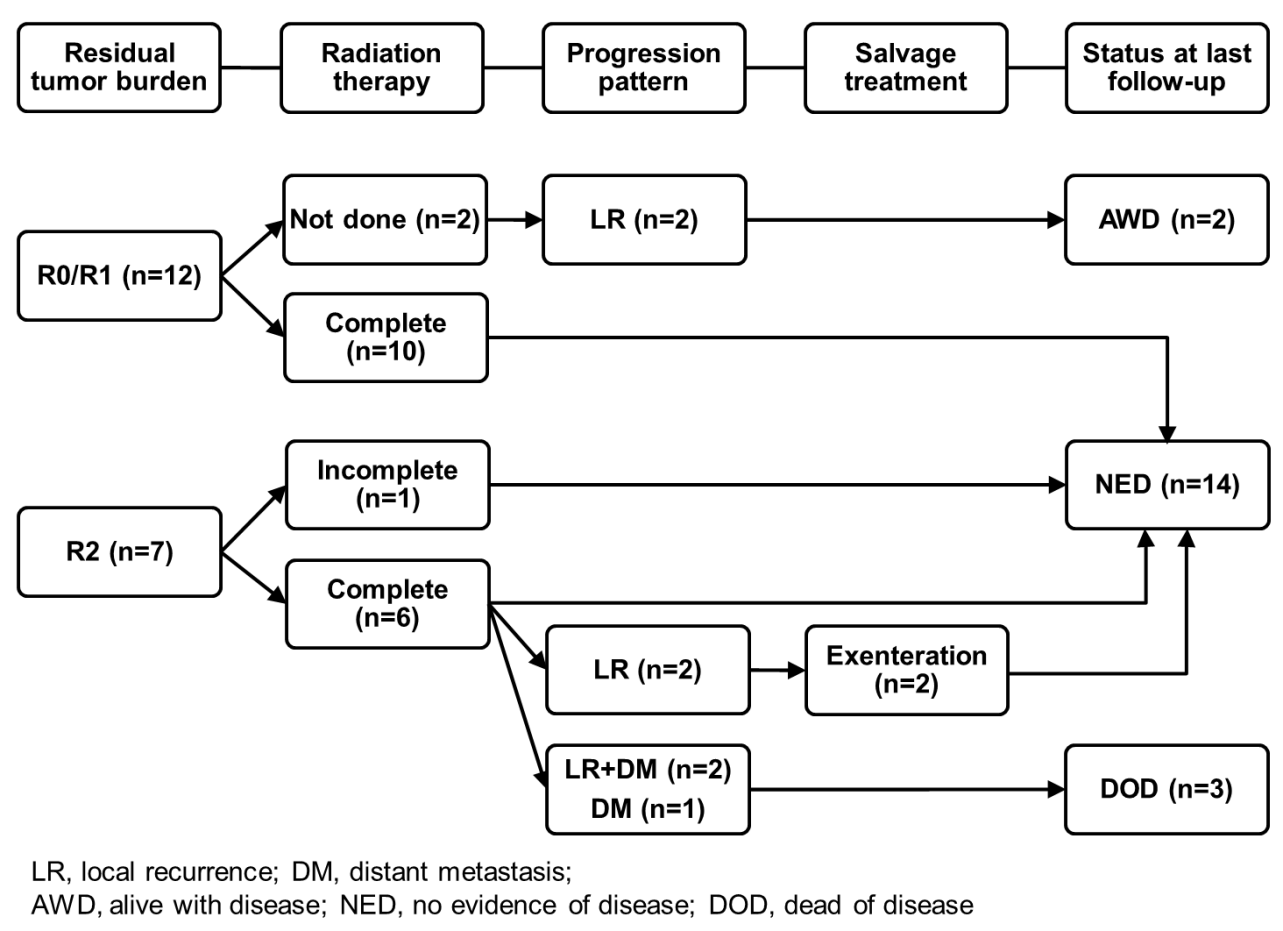
Treatment outcomes and patterns of progression

**Figure 2:**
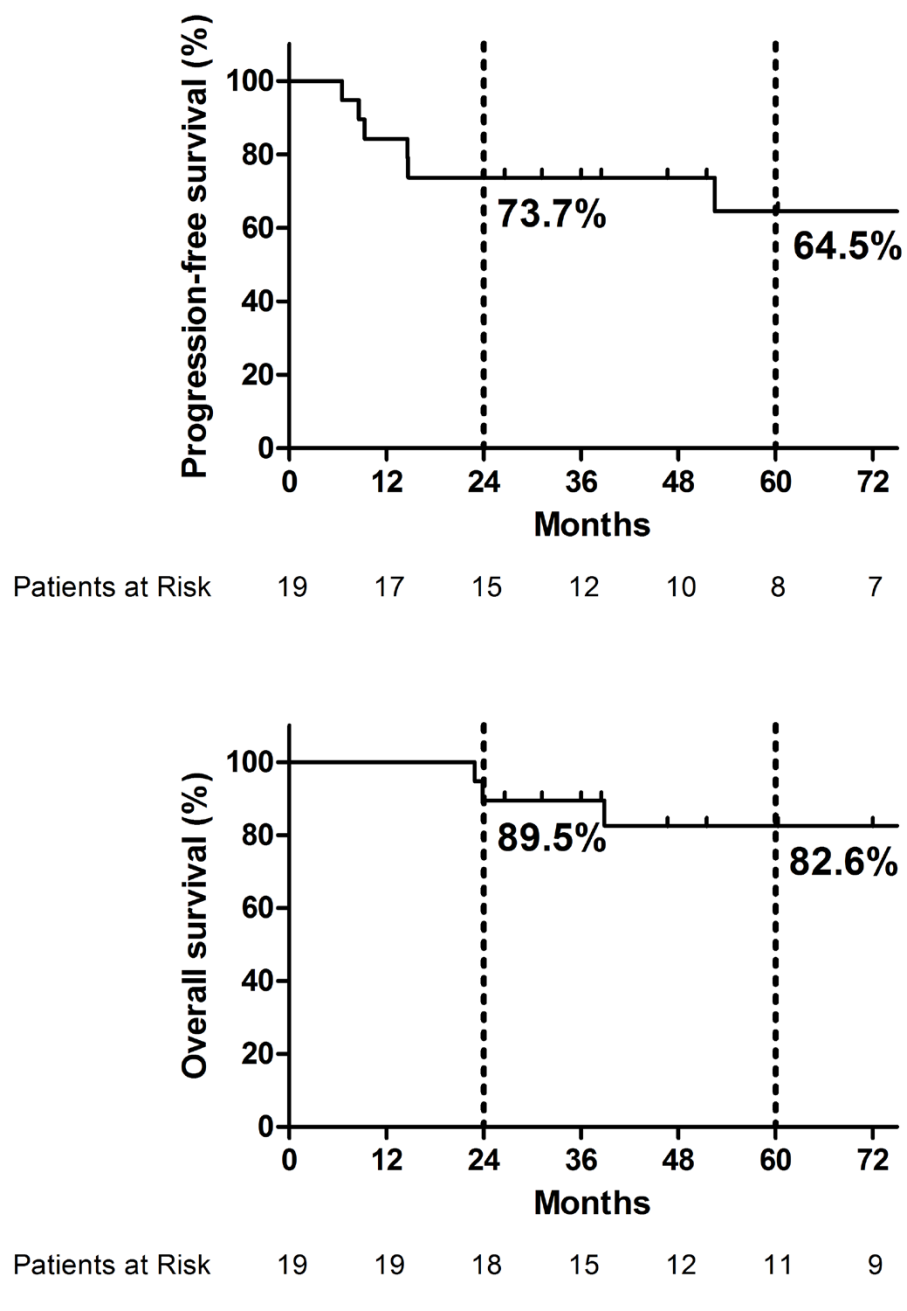
Progression-free survival and overall survival of all patients

During the median follow-up of 57.5 months (25.9∼212.0 months) in all patients, seven (36.8%) developed progression and three (15.8%) died. The patterns of progression were local recurrence in four patients (21.1%), distant metastasis in one (5.3%) and combined local recurrence and distant metastasis in two (10.5%), respectively. The median time to any type of progression was 14.6 months (6.5∼191.7 months) (Table 3 and Figure 1). The time to distant metastasis was relatively short (8.6, 9.3 and 14.6 months), while that to local recurrence was wide in range (6.5, 14.7, 52.5 and 191.7 months). The progression-free survival (PFS) and overall survival (OS) rates at 2- and 5-years of all patients were 73.7% and 64.5%, and 89.5% and 82.6%, respectively (Figure 2).

**Table 2:** Patients’ characteristics

**Characteristics**	**Number of patients**	
Age		
<40 years		7 (36.8%)
≥40 years		12 (63.2%)
Gender		
Male		9 (47.4%)
Female		10 (52.6%)
Clinical T stage		
T1		8 (42.1%)
T2		1 (5.3%)
T4		10 (52.6%)
4b	6	
4c	4	
Tumor size		
<3.0 cm		7 (36.8%)
≥3.0 cm		12 (63.2%)
Type of surgery		
Orbital exenteration		3 (15.8%)
Gross total resection		11 (57.9%)
Biopsy only		5 (26.3%)
Residual tumor burden		
R0		1 (5.3%)
R1		11 (57.9%)
R2		7 (36.8%)
Radiation therapy		
Not done		2 (10.5%)
Incomplete		1 (5.3%)
Done		16 (84.2%)

Abbreviations: R0, complete resection with no microscopic residual tumor; R1, complete resection with no gross residual tumor but microscopic involvement of the resection margin on pathological report; R2, partial resection with gross residual tumor or radiographic evidence of gross residual tumor

**Table 3:** Patients’ characteristics and clinical outcomes

Case	Age (years)/Gender	Tumor size	Clinical T stage	Subtype	Perineural invasion	Initial treatment	Residual burden	Progression pattern	Salvage treatment	Status at last follow-up^†^
1	17/F	2.0 cm	T1	Cribriform	Not known	Resection + RT	R1	--	--	NED at 57.5 months
2	40/M	3.5 cm	T2	Cribriform	Present	Resection + RT	R1	--	--	NED at 199.6 months
3	24/F	4.0 cm	T2	Cribriform	Present	Resection + RT	R1	--	--	NED at 78.0 months
4	26/F	2.5 cm	T2	Cribriform	Present	Resection + RT	R1	--	--	NED at 34.6 months
5	46/M	2.5 cm	T2	Cribriform	Present	Resection + RT	R1	--	--	NED at 26.7 months
6	45/M	3.0 cm	T2	Cribriform	Present	Resection + RT	R1	--	--	NED at 21.9 months
7	26/F	2.6 cm	T2	Cribriform	Present	Resection	R1	LR at 14.7 months	Not done	AWD at 59.1 months (44.4 months)
8	30/F	3.8 cm	T2	Solid	Not known	Biopsy + RT	R2	LR at 6.5 months	Exenteration	NED at 111.4 months (104.9 months)
9	43/F	3.0 cm	T2	Cribriform	Not known	Resection + RT	R2	LR at 52.5 months	Exenteration	NED at 138.8 months (86.3 months)
10	39/F	3.0 cm	T4b	Not known	Not known	Exenteration + RT	R0	--	--	NED at 190.1 months
11	43/F	3.2 cm	T4b	Cribriform	Present	Exenteration + RT	R1	--	--	NED at 33.0 months
12	55/M	2.5 cm	T4b	Cribriform	Present	Resection + RT	R1	--	--	NED at 199.6 months
13	64/M	3.5 cm	T4b	Cribriform	Not known	Resection + RT	R1	--	--	NED at 133.7 months
14	40/M	3.5 cm	T4b	Tubular	Present	Resection	R1	LR at 191.7 months	Not done	AWD at 212.0 months (20.3 months)
15	44/F	2.5 cm	T4b	Cribriform	Not known	Biopsy + RT	R2	DM (Lung, Bone) at 14.6 months	Chemotherapy	DOD at 38.9 months (24.3 months)
16	61/M	3.0 cm	T4c	Tubular	Present	Exenteration + RT*	R2	--	--	NED at 22.7 months
17	33/M	2.5 cm	T4c	Tubular	Not known	Biopsy + RT	R2	--	--	NED at 25.9 months
18	40/F	4.0 cm	T4c	Solid	Not known	Biopsy + RT	R2	LR+DM (Bone) at 9.3 months	None	DOD at 23.9 months (14.6 months)
19	56/M	4.1 cm	T4c	Not known	Not known	Biopsy + RT	R2	LR+DM (Bone) at 8.6 months	Chemotherapy	DOD at 22.9 months (14.3 months)

Abbreviations: RT, radiation therapy; R0, complete resection with no microscopic residual tumor; R1, complete resection with no gross residual tumor but microscopic involvement of the resection margin on pathological report; R2, partial resection with gross residual tumor or radiographic evidence of gross residual tumor; LR, local recurrence; DM, distant metastasis; NED, no evidence of disease; AWD, alive with disease; DOD, dead of disease

* This patient did not complete RT because of wound problem.

† Durations in parentheses are the intervals from the time of progression till the last follow-up.

Among 12 patients who underwent R0/1 resection, no recurrence occurred in ten patients (83.3%), who received RT, and two (16.7%), who refused RT, developed local recurrence in 14.7 and 191.7 months from the initial surgery, respectively (case 7 and 14 in Table 3). Though these two patients with local recurrence refused further salvage treatment, they were still alive with slowly progressing disease until the last follow-up at 44.4 and 20.3 months, respectively. Consequently, there was no one who died among the patients who underwent R0/1 resection until the current analysis. Meanwhile, among seven patients who underwent R2 resection for any reason, five (71.4%) developed progression: local recurrence in two (28.6%); distant metastasis in one (14.3%); and combined local recurrence and distant metastasis in two (28.6%), respectively. Two patients, who developed local recurrence in 6.5 and 52.5 months, initially underwent RT following biopsy alone and partial tumor removal for T2 disease. These two were successfully salvaged by orbital exenteration (104.9 and 86.3 months since the time at local recurrence, case 8 and 9 in Table 3). However, all three patients who initially underwent RT following biopsy alone for T4 disease (T4b in one and T4c in two) developed distant metastasis and died of disease (case 15, 18 and 19 in Table 3).

Review of the pathological slide was possible in 17 patients: 12 (70.6%) had cribriform pattern; three (17.6%) had tubular pattern; and two (11.8%) had solid pattern, respectively. Progression occurred in three patients among 12 having cribriform pattern (25.0%), one among three having tubular patterns (33.3%), and both two having solid pattern (100%), respectively. Presence of perineural invasion was recorded in ten patients (52.9%), among who two (20.0%) developed local recurrence following R1 resection without receiving RT. The remaining eight patients were known to have no evidence of disease following surgery and RT.

On univariate analysis, the presence of intracranial extension (T4c) was associated with significantly poorer OS at 5 years than the others (91.7% *vs.* 50.0%, *P=.0117*). And the presence of gross residual tumor burden following surgery (R2) was associated with significantly poorer PFS at 2 years (91.7% *vs.* 42.9%, *P=.0012*) and OS at 5 years (100% *vs.* 53.6%, *P=.0149*) than the others, respectively (Table 4).

**Table 4:** Univariate analysis for progression-free survival and overall survival

Characteristics (number)	Progression-free survival at 2 years	P-value	Overall survival at 5 years	*P*-value
Age				
<40 years (7)	71.4%	0.7175	100%	0.1744
≥40 years (12)	75.0%		72.9%	
Gender				
Male (9)	88.9%	0.1832	88.9%	0.6600
Female (10)	60.0%		77.1%	
Clinical T stage				
T1/2 (9)	77.8%	0.8945	100%	0.0804
T4 (10)	70.0%		66.7%	
Intracranial Extension				
No (15)	80.0%	0.1787	91.7%	0.0117
Yes (4)	50.0%		50.0%	
Tumor size				
<3.0 cm (7)	71.4%	0.6863	75.0%	0.9373
≥3.0 cm (12)	75.0%		83.3%	
Residual tumor burden				
R0/1 (12)	91.7%	0.0012	100%	0.0149
R2 (7)	42.9%		53.6%	
Radiation therapy				
Not done (3)	66.7%	0.4673	100%	0.4074
Done (16)	75.0%		78.8%	

Abbreviations: R0, complete resection with no microscopic residual tumor; R1, complete resection with no gross residual tumor but microscopic involvement of the resection margin on pathological report; R2, partial resection with gross residual tumor or radiographic evidence of gross residual tumor

## DISCUSSION

The 7th edition AJCC Staging Manual has subdivided T4 stage based on the extent of tumor involvement from the periosteum (T4a), orbital bone (T4b), and to the adjacent structures (T4c) (Table 1). The prognostic accuracy of T stage by the 7th edition AJCC manual was addressed by El-Sawy et al. [10], who analyzed 18 consecutive patients. Even though the El-Sawy's study and the current study included the similar number of patients with lacrimal ACC who underwent curative treatment, there were a few differences. First, the proportion of T4 disease was higher in the El-Sawy's study than the current study (14/18, 77.8% *vs.* 10/19, 52.6%), and there was no case with T4c in the El-Sawy's study, while the current study included four cases with T4c. Second, the actually applied treatment modality was different: all patients except one underwent orbital exenteration in the El-Sawy's study and all received RT post-operatively, while all except three underwent globe-preserving surgery and 17 received RT post-operatively in the current study. Third, the failure patterns consequently were quite different: the component of distant metastasis was more frequent in the El-Sawy's study (8/18, 44.4% *vs.* 3/19, 15.8%), while that of local failure, however, was reversed (3/18, 16.7% *vs.* 6/19, 31.6%). These might naturally be explained by the differences in the patients’ proportion having T4c disease and the aggressiveness of surgical procedures. Both studies, however, have in common that there was a tendency of more frequent failure, though not significant, along with the increasing T stage.

With respect to the treatment policy for lacrimal ACC, the extent of surgical resection (orbital exenteration *vs.* globe-preserving surgery) has been an unsolved issue as of yet. According to a retrospective multicenter analysis, globe-preserving surgery seemed quite effective in treating the patients having T1-2 disease [8]. In the current study, among nine patients having T1-2 disease, all underwent globe-preserving surgery, and three developed local recurrence. Two patients of these three were successfully salvaged by orbital exenteration following local recurrence and none among nine died until the current analysis. Three patients with local recurrence included two patients who had gross residual disease following R2 resection and one who did not receive RT following R1 resection. When performing globe-preserving surgery in the patients with T1-2 disease, more careful attention in order not to leave gross residual might be necessary and the addition of RT should be strongly recommended.

Orbital exenteration should be considered in order to achieve negative resection margins in treating the patients with T3 or higher disease, which is not infrequently hesitated mainly in consideration of poor cosmesis, compromised self-esteem and social integrity. In the current study, though ten patients were candidates for orbital exenteration as having T4 disease, only three underwent orbital exenteration, while seven did globe-preserving surgery (three gross resection and four biopsy only, respectively). Three patients among four, who underwent biopsy, had T4c disease by virtue of intracranial extension. Three patients developed distant metastasis within relatively short follow-up period. Moreover, one patient with T4c disease suffered from wound problem following upfront orbital exenteration and had to terminate RT at its early course. Two patients who developed local recurrence were successfully salvaged following orbital exenteration. Overall, though more than one-third of all patients (7/19, 36.8%) were classified as R2 resection, more than two-thirds of the alive patients (11/16, 68.8%) could keep the intact globe. Considering these observations, the decision on performing upfront orbital exenteration should be made cautiously considering the disease extent and the feasibility of R0/1 resection through in-depth discussion with the patients.

When analyzing the clinical outcomes with the special focus on the residual tumor burden following surgery, authors found that gross residual was a significantly important determinant on both PFS and OS. Most patients who underwent R2 resection experienced progression (Figure 1), and as a result, the rates of PFS at 2 years and OS at 5 years were 42.9% and 53.6%, respectively (Table 4). Among those who received RT following R0/1 resection, no progression was observed. On the contrary, two patients, who refused RT despite following R1 resection, however, experienced local recurrence. Based on these, authors would speculate that the gross residual following surgery was a very important prognostic determinant and that the addition of RT was able to reduce local recurrence if not having gross residual burden.

The patients with cribriform pattern tended to show better clinical outcomes than the others having tubular or solid pattern in the current study. Among ACC, solid subtype has been known to be one of the poor prognostic factors [11], which was affirmed in El-Sawy's study [10], but was not conclusive in the current study, as there were only two patients who had solid subtype. Addition of RT seemed to have been effective with respect to local control in patients having perineural invasion.

In order to overcome the issue of poor clinical outcomes, a few alternative approaches may be considered. Tse et al. retrospectively analyzed 19 patients who were treated with neoadjuvant intra-arterial cytoreductive chemotherapy, orbital exenteration and post-operative RT [12]. They employed intra-arterial chemotherapy in order to enhance the completeness of surgical resection with the potential of saving the globe, and observed that the cumulative OS and disease-free survival rates at 10 years were 100% and 100%, respectively. In case when radical surgery is not feasible either because of the patients’ medical condition or because of the concern on the post-surgical cosmetic outcome, radiation dose escalation highly focused to the target region using the advanced RT techniques could be considered.

Fast neutron therapy, by virtue of higher relative biological effectiveness than photons, was found to improve local control in a randomized study of patients with unresectable salivary gland tumors [13]. Recent report on neutron therapy (with or without radiosurgery boost) to 11 lacrimal ACC patients suggested a promising long-term local control (80% at 5 years), however, delayed occurrence of disease progression and severe eye and neural complication seemed to be a significant obstacle [14].

Particle beams, including protons and heavy ions, can offer dramatic dose fall-off at the interface between the target volume and the normal tissues by virtue of Bragg-peak phenomenon. Takagi et al. [15] retrospectively reviewed on 80 patients with ACC in various head and neck regions who were treated by either protons or carbon ions. They could achieve quite promising clinical outcomes: the 5-year rates of OS, PFS and local control were 63%, 39% and 75%, respectively. However, they experienced frequent local failures if the patients had T4 disease or inoperable lesion and grade 3 or higher late toxicities if dose was greater than 72 GyE (24/56, 43%). They suggested the importance of optimizing treatment planning to reduce the incidence and severity of late toxicities while achieving high level local control. Planning of ideal RT always starts from delineating the target volume and the normal structures as accurately as possible. Computed tomography (CT) and magnetic resonance imaging (MRI) are complementary imaging modalities in the localization and characterization of lacrimal fossa lesions [16]. CT has the advantages of demonstrating bone destruction and calcification while MRI is the preferred in assessing perineural invasion and intracranial extension. MRI usually provides optimal soft tissue contrast that allows better demonstration of the extent of soft tissue abnormality than CT. In this regard, use of MRI in addition to CT in delineating the target volume is highly recommended, especially when tumors are very close to and/or actually invading the skull base.

## CONCLUSIONS

Based on the current observations, two important summaries could be made: first, globe-preserving surgery followed by RT seemed an optimal treatment strategy for those having T1-2 disease; second, the addition of RT following R1 resection could decrease the risk of progression, but not following R2 resection. These, however, naturally leave a dilemma on the optimal treatment strategy for those who are potential candidates for orbital exenteration based on the tumor extent. Whenever possible, a careful attention should to be paid in order not to leave gross residual at the time of surgery. In addition, an effort to escalate the radiation dose to the focal region of risk with the guaranteed safety would be highly desirable.

## MATERIALS AND METHODS

From January 1997 to April 2013, 22 patients were treated with curative intent for lacrimal ACC at our institution. After excluding three patients who had history of recurrence, 19 formed the basis of the current study. After approval by the Institutional Review Board (2014-06-001), each patient's medical records, histological sections and radiographic images were thoroughly reviewed. Stage assignment was done based on the 7th edition of the AJCC Staging Manual. All available pathologic slides were reevaluated by two experienced pathologists.

All patients underwent upfront surgical intervention and RT was recommended in 4∼6 weeks of surgery. RT target volume delineation was to include the tumor bed and/or gross residual tumor based on the surgical records, pathological reports, and post-operative imaging. No one received planned chemotherapy in relation to the current treatment.

The first follow-up evaluation, including physical examination and imaging study, was performed in 3∼4 weeks of planned treatment completion and subsequent follow-up evaluations were regularly scheduled at 3∼4 months’ interval for the first two years and at 6∼12 months’ interval thereafter. Progression was defined as the appearance of new lesion or increasing size of existing lesion. The durations of PFS and OS were defined as the time from the surgery till the dates of disease progression and death from any causes or the last follow-up. The rates of PFS and OS were calculated by Kaplan-Meier method and their comparisons were done by the log-rank test. Statistical analyses were performed using SAS software version 9.1.3.
